# Productive and economic effects of adding *Bacillus amyloliquefaciens* CECT 5940 to bread waste-based diets in laying hens

**DOI:** 10.14202/vetworld.2025.969-975

**Published:** 2025-04-23

**Authors:** Albertina Felizardo Manteiga, Abilio Paulo Changule, Nilza Jorge Manjate, Dercia Hilario Magaia, Florentina Domingos Chilala, Leonel Antonio Joaquim, Eunice Justino Chivale, Filomena dos Anjos, Manuel Garcia-Herreros, Custódio Gabriel Bila

**Affiliations:** 1 Department of Animal and Public Health, Faculty of Veterinary Medicine, Eduardo Mondlane University, Maputo 1304, Mozambique; 2 Center for Genetic Resources and Animal Assisted Techniques, Directorate of Animal Science, Agricultural Research Institute of Mozambique, Matola 1410, Mozambique; 3 Department of Research and Development, Intermed Mozambique Lda, Maputo 1304, Mozambique; 4 Department of Pharmacy, School of Pharmacy, Federal University of Ouro Preto, Ouro Preto 35402-278, Brazil; 5 Department of Biotechnology and Animal Medicine of the Amazon Veterinary Medicine Institute, Federal University of Pará, Belém 66075-110, Brazil; 6 Department of Animal Production, Agricultural Research Institute of Mozambique, Angonia 2306, Mozambique; 7 Department of Animal Science, Institute of Veterinary Medicine, Federal University of Pará, Castanhal 68746-360, Brazil; 8 Department of Animal Production and Food Technology, Faculty of Veterinary Medicine, Eduardo Mondlane University, Maputo 1304, Mozambique; 9 Section of Animal Nutrition, Faculty of Veterinary Medicine, Eduardo Mondlane University, Maputo 1304, Mozambique; 10 Department of Animal Production Systems, National Institute for Agricultural and Veterinary Research, Santarém 2005-424, Portugal; 11 CIISA-AL4AnimalS, Faculty of Veterinary Medicine, University of Lisbon, 1300-477 Lisbon, Portugal; 12 Center of Excellence in Agri-Food Systems and Nutrition - Eduardo Mondlane University, Maputo 257, Mozambique

**Keywords:** *Bacillus amyloliquefaciens*, bread waste, egg production, feed cost, laying hens, poultry nutrition, probiotic

## Abstract

**Background and Aim:**

The high cost and limited availability of maize in poultry production necessitate the exploration of alternative feed sources. Bread waste (BW), a nutrient-rich by-product, offers a sustainable substitute. However, concerns regarding its protein degradation during processing and limited research in laying hens restrict its broader application. Probiotics such as *Bacillus amyloliquefaciens* CECT 5940 may enhance nutrient utilization and economic viability when combined with BW. This study aimed to evaluate the effects of partially replacing maize meal with BW, with or without the inclusion of *B. amyloliquefaciens* CECT 5940, on the productive performance and economic outcomes in laying hens.

**Materials and Methods:**

Sixty 52-week-old Lohmann Brown laying hens were randomly assigned to one of three treatments (n = 20 per treatment; 4 replicates of 5 hens each): (T1) basal diet; (T2) basal diet with 20% BW replacing maize; and (T3) basal diet with 20% BW and 0.8% *B. amyloliquefaciens* CECT 5940. Performance parameters and economic indicators, including feed intake, egg production, feed conversion ratios, gross revenue, contribution margin, and break-even point, were assessed over 45 days.

**Results:**

No significant differences (p > 0.05) were observed in laying rate, egg mass, feed conversion per dozen eggs, or live weight across treatments. Economically, substituting 20% of maize with BW significantly reduced feed cost per kilogram and egg production cost per unit and per dozen (p < 0.05). The inclusion of *B. amyloliquefaciens* further enhanced the profitability index, gross value added, and reduced the break-even point compared to T1 and T2 (p < 0.05).

**Conclusion:**

Replacing 20% of maize with BW, with or without *B. amyloliquefaciens* CECT 5940, maintains productive performance in laying hens while significantly improving economic efficiency. This strategy offers a viable and sustainable alternative to conventional feed formulations in poultry production.

## INTRODUCTION

Laying hen production plays a vital role in delivering animal protein that is both nutritionally rich and economically accessible [1]. Nonetheless, the escalating cost of maize has led to increased feed expenses, rendering certain farming operations unprofitable and adversely impacting the egg industry [2]. This surge in maize prices is primarily due to ongoing challenges such as droughts, flooding, disease outbreaks, and limited investment in grain cultivation. Consequently, there is a pressing need to identify alternative feed resources [3] capable of partially or fully substituting maize meal, especially during periods of shortage [4].

Among such alternatives, bakery waste has demonstrated considerable promise [5], offering cost-saving advantages while sustaining poultry productivity and consumer satisfaction. Although bakery waste has been explored as a non-conventional energy source in poultry diets, specific investigations into its incorporation into laying hen feed are still limited, highlighting a notable gap in the literature that this study intends to address [6]. In particular, bread waste (BW) – a widely available, inexpensive, and commonly consumed bakery product – has received minimal attention in the context of laying hen nutrition [7]. Prior research by Truong *et al*. [6] and Yadav and Jha [8] confirmed the economic feasibility of using BW as a partial maize substitute in broiler feed. Despite these encouraging findings, comprehensive economic assessments of BW inclusion in laying hen diets remain scarce.

Moreover, the continued application of subtherapeutic antibiotics in poultry feed to promote growth and prevent disease has raised significant concerns about antimicrobial resistance, which poses risks to both animal and public health [9]. As a result, many nations have imposed restrictions or bans, prompting the search for alternative solutions. Probiotics have emerged as a viable substitute, enhancing gut health in broilers by modulating intestinal microbiota [10, 11]. Among these, *Bacillus* species are particularly promising due to their ability to inhibit pathogens, enhance growth performance, improve nutrient digestibility, support immune function, and maintain gut integrity [12]. Specifically, *Bacillus amyloliquefaciens* CECT 5940 has yielded positive results in broilers as a direct-fed microbial, both independently and when combined with bacitracin methylene disalicylate, particularly under enteric pathogen challenge [9, 10]. However, there is limited evidence regarding its application in laying hen production, and the continued reliance on antibiotics in this sector remains a concern.

Despite the growing interest in utilizing bakery by-products as alternative feed sources in poultry nutrition, empirical data on their application in laying hen diets remains limited. While BW has shown promise in broiler production due to its affordability and nutritional composition, its impact on the productive performance and economic outcomes of laying hens is underexplored. Moreover, studies investigating the synergistic effects of BW and probiotic supplementation, particularly *B. amyloliquefaciens* CECT 5940, are virtually absent in the literature. The existing body of work primarily focuses on broilers, leaving a critical knowledge gap concerning the applicability and economic viability (Vb) of these interventions in layer production systems. Furthermore, while the replacement of maize meal with BW has been suggested as a cost-reduction strategy, comprehensive assessments integrating performance metrics and profitability indices are lacking, particularly under practical production conditions in resource-limited settings.

The present study aimed to evaluate the effects of partially replacing maize meal with BW, with or without the inclusion of *B. amyloliquefaciens* CECT 5940, on the productive performance and economic efficiency of laying hens. Specifically, the study assessed key zootechnical parameters, including feed intake, egg production rate, egg mass (EM), and feed conversion ratios, alongside detailed economic analyses such as feed cost per unit, cost of egg production, gross revenue, contribution margin, and break-even point. By addressing both biological performance and profitability, this study seeks to provide a scientifically grounded, sustainable alternative to conventional maize-based diets, thereby contributing to the advancement of cost-effective poultry production systems.

## MATERIALS AND METHODS

### Ethical approval

This study was approved by the Ethics Committee of the Eduardo Mondlane University (Approval Number: 980FAVET, June 12, 2023).

### Study period and location

The study was conducted from July to September 2023. The experimental trial was conducted at the animal facility of InterMed Mozambique Lda, located in the Marracuene district of Maputo Province, Mozambique (latitude: ~25.8976° S; longitude: ~32.4059° E), at an elevation of approximately 26 m above sea level. A total of 60 Lohmann Brown laying hens, which had been laying eggs for 30 weeks before the study, were utilized in a 45-day experiment. The hens were housed in a battery cage system situated over a deep pit, with access to both natural and artificial lighting and natural ventilation.

### Experimental design

The hens were randomly allocated into three groups of 20 birds each, with four replicates per group (n = 4), in a completely randomized design. The treatment groups were as follows: T1 – Basal diet; T2 – Basal diet with 20% BW replacing 20% maize meal; and T3 – Basal diet with 20% BW plus 0.8% *B. amyloliquefaciens* CECT 5940 (Evonik, Essen, Germany), replacing 20% maize meal. The composition and calculated nutrient content of the experimental diets are presented in Table 1. Both the basal feed and BW were sourced locally. All birds were maintained under uniform management conditions. Water was provided *ad libitum*, and feed was offered at 120 g/hen/day. Leftover feed was weighed daily to calculate feed intake. All experimental groups received identical handling throughout the trial period.

**Table 1:** Composition of experimental diets.

Ingredients	Calculated composition (kg)		

	T1	T2	T3
Maize meal	62.50	50.00	50.00
Soybean meal	12.20	12.20	12.20
Wheat bran	16.95	16.95	16.15
Cotton-seed cake meal	5.00	5.00	5.00
Bread waste	0.00	12.50	12.50
*Bacillus amyloliquefaciens* CECT 5940	0.00	0.00	0.80
Trace mineral premi×^1^	3.00	3.00	3.00
Vitamin premi×^2^	0.10	0.10	0.10
Dicalcium phosphate	0.25	0.25	0.25
Total	100	100	100
Calculated nutrient composition			
Energy (kcal/kg)	3,130.98	3,379.54	3,379.54
Protein	14.60	14.50	14.50
Fiber	3.95	2.91	2.91
Ether extract	3.31	2.88	2.88
Lysine	2.31	2.29	2.29
Methionine	1.60	1.58	1.58
Calcium	4.00	4.04	4.04
Phosphorus	0.40	0.70	0.70

^1^Trace mineral premix provided per kilogram of diet: Mn, 80 mg; Fe, 60 mg; Zn, 60 mg; Cu, 5 mg; Co, 0.2 mg; I, 1 mg; Se, 0.15 mg; Ca, 446.9 mg. ^2^Vitamin premix provided per kilogram of diet: vitamin A, 12,000 IU; cholecalciferol, 2000 IU; vitamin E, 35 IU; vitamin k_3_, 5 mg; thiamin, 3 mg; riboflavin, 6 mg; niacin, 20 mg; Ca-d-pantothenate, 6 mg; pyridoxine, 5 mg; vitamin B_12_, 15 g, folacin, 0.75 mg, D-biotin, 45 g, choline chloride, 125 mg; vitamin C, 50 g

Initial body weight and egg production rates were documented at the onset of the experiment and monitored on a weekly basis. Daily records were maintained for egg production, soft-shelled eggs, egg weight, and mortality, while weekly feed consumption was also tracked. Parameters such as feed intake, egg production ratio, soft-shelled egg ratio, feed conversion per EM (FC/EM), feed conversion per dozen eggs (FC/dz), and mortality rates were calculated weekly in accordance with the methodology of Novela *et al*. [4].

### Economic analyses

The economic indicators evaluated included feed cost, egg production cost per unit and per dozen, gross revenue, profitability index, gross value added, and break-even point. These were calculated following the methods outlined by Andriani *et al*. [13] and Egbetokun and Obisesan [14]. Feed cost estimation incorporated the individual and total ingredient prices used in each treatment group. The cost per egg was derived by dividing the total production cost by the number of eggs produced, while the cost per dozen eggs was based on the quantity of feed required to produce a dozen eggs relative to feed cost. Gross revenue was calculated based on the number of eggs produced and their respective unit sales prices. The gross value added was determined by subtracting the total feed cost from the total sales revenue. The profitability index represented the proportion of income generated, and the break-even point indicated the number of eggs required to cover feed costs.

### Statistical analysis

Data were subjected to a one-way analysis of variance using the Statistical Package for the Social Sciences software version 25 (IBM Corp., NY, USA). Differences between treatment means were compared using Tukey’s test at a 5% level of significance.

## RESULTS

### Aaa

**Effects of *B. amyloliquefaciens* CECT 5940 supplementation on the performance of laying hens fed BW-based diets**

The outcomes of substituting 20% of maize meal with BW, with or without the addition of *B. amyloliquefaciens* CECT 5940, are summarized in Table 1. Relative to the basal diet (T1), the inclusion of 20% BW (T2) had no significant impact on laying hen live weight (LW), egg-laying rate (LR), EM, FC/dz, or Vb (p > 0.05). Although these differences were not statistically significant (p > 0.05), hens receiving the BW diet supplemented with *B. amyloliquefaciens* CECT 5940 (T3) demonstrated numerically superior values for body weight, LR, EM, and FC/dz.

Table 2 presents the economic effects of partially replacing maize meal with BW, with or without *B. amyloliquefaciens* CECT 5940 supplementation (T3), on the cost of feed production per kilogram, total feed cost, and production cost per egg and per dozen eggs.

**Table 2:** Effects of partial replacement of corn with or without the addition of *Bacillus amyloliquefaciens* CECT 5940.

Parameter	T1	T2	T3
LW	1473	1436	1571
FC (kg)	10.80	10.80	10.80
LR (%)	66.6 ± 12.94	61.11 ± 18.41	72.89 ± 8.29
EM	0.27 ± 0.09	0.32 ± 0.11	0.34 ± 0.07
FC/EM (kg)	2.10 ± 1.12	1.62 ± 0.60	1.47 ± 0.39
FC/dz (kg)	1.12 ± 0.25	1.27 ± 0.39	0.99 ± 0.47
Vb (%)	100	100	100

Mean ± standard deviation. No significant differences were observed among treatments with the same parameter (p > 0.05). LW=Live weight of the layers, FC=Feed consumption, LR=Laying rate, EM=Egg mass, FC/EM=Feed conversion per egg mass, FC/dz=Feed conversion per dozen, Vb=Viability of the layers, T1=Commercial feed, T2=Inclusion of 20% bread flour, T3=Inclusion of 20% bread flour with probiotic *Bacillus amyloliquefaciens* CECT 5940

### Cost-benefit analysis of *B. amyloliquefaciens* CECT 5940 inclusion in BW-based diets

The data revealed a statistically significant reduction in feed production cost, decreasing from $0.55 in T1 to $0.34 in T3. Similarly, the cost of feed per bird dropped from $5.96 in T1 and $3.77 in T2 to $3.71 in T3. This downward trend was also observed in egg production costs: the cost per egg fell from $0.05 in the control group to $0.02 in the BW treatments, and the cost per dozen eggs declined from $0.62 to $0.35 (Table 3).

**Table 3:** Economic evaluation of corn replacement with 20% inclusion of bread waste and *Bacillus amyloliquefaciens* supplementation (CECT 5940).

Parameters	T1	T2	T3
Cost of producing feed ($/kg)	0.55^a^	0.35^b^	0.34^b^
Cost of feed ($)	5.96^a^	3.77^b^	3.71^b^
Cost of egg production ($)	0.05 ± 0.01^a^	0.02 ± 0.20^b^	0.02 ± 0.18^b^
Cost of egg production/Dz ($)	0.62 ± 0.14^a^	0.35 ± 0.04^b^	0.35 ± 0.03^b^

Means ± standard deviation. Different letters within the same row (a, b) represent statistical differences among treatments within the same parameter (p ≤ 0.05); T1=Commercial feed, T2=Inclusion of 20% bread waste, T3=Inclusion of 20% bread waste with probiotic *Bacillus amyloliquefaciens* CECT 5940. $=United States Dollar

Table 4 illustrates the impact of BW and *B. amyloliquefaciens* CECT 5940 inclusion on economic profitability, evaluated through gross revenue, gross value added, profitability index, contribution margin, and break-even point. Replacing maize with 20% BW decreased the gross revenue per product unit from $9.39 (T1) to $8.61 (T2), whereas supplementation with *B. amyloliquefaciens* CECT 5940 increased it by approximately $0.81 (T3).

**Table 4:** Effects of partial replacement of maize meal with leftover bread and the addition of *Bacillus amyloliquefaciens* CECT 5940 on economic profitability.

Treatment	Gross revenue	Gross value added ($)	Profitability index (%)	Contribution margin ($)	Contribution margin (%)	Break-even point ($)
T1	9.39 ± 1.82^a^	3.47 ± 1.82^a^	0.54 ± 0.22^a^	0.67 ± 0.07^a^	67.36 ± 7.40^a^	8.88 ± 1.10^a^
T2	8.61 ± 2.59^a^	5.00 ± 2.59^ab^	0.86 ± 0.22^ab^	0.77 ± 0.07^ab^	77.39 ± 7.03^ab^	4.69 ± 0.44^b^
T3	10.20 ± 1.24^a^	6.47 ± 1.24^b^	0.98 ± 0.07^b^	0.81 ± 0.02^b^	81.60 ± 2.03^b^	4.58 ± 0.11^b^

Means ± standard deviation. Different letters within the same column (a, b) represent statistical differences among treatments within the same parameter (p < 0.05). T1=Commercial feed, T2=Inclusion of 20% bread waste, T3=Inclusion of 20% bread waste with probiotic *Bacillus amyloliquefaciens* CECT 5940. $=United States Dollar

Among the three treatments, T3 yielded the highest values for gross value added, profitability index, and contribution margin, while T1 showed the lowest. Although differences among treatments for these economic parameters were not statistically significant (p > 0.05), *B. amyloliquefaciens* CECT 5940 supplementation resulted in a statistically significant improvement in both the contribution margin and break-even point when compared with T1 and T2 (Table 4).

## DISCUSSION

Our findings confirm that processed food products originally intended for human consumption but discarded as waste can serve as valuable alternatives in laying hen diets, in addition to their economic advantages. Bakery waste is rich in nutrients such as wheat flour, corn, sugar, and vegetable oil, rendering it a suitable energy source for poultry feed [15]. In this study, performance indicators – including LW, FC/EM, and FC/dz – were only minimally affected by the inclusion of *B. amyloliquefaciens* CECT 5940 when 20% of maize meal was replaced with BW. *Bacillus* species, along with other probiotics, are widely used to enhance animal performance and health [16]. The observed improvements in LW, FC/EM, and FC/dz associated with *B. amyloliquefaciens* CECT 5940 supplementation may be attributed to the production of digestive enzymes such as proteases, amylases, and cellulases [17].

Earlier studies on protein digestibility in poultry have indicated that a significant amount of dietary protein passes through the gastrointestinal tract without complete digestion [18]. Consequently, the addition of exogenous enzymes, such as proteases, has been proposed to enhance protein digestibility in laying hens. *Bacillus* spp. is known to stimulate the production of these enzymes, which aid in the breakdown of crude protein and essential amino acids, thereby sustaining optimal performance in commercial diets. Moreover, the supplementation of laying hen diets with exogenous proteases has been shown to maintain egg production rates under nutritionally deficient conditions [19] and to improve feed conversion ratios in nutritionally adequate diets [20]. However, in the present study, no statistically significant differences were detected compared to the control group (T1). This aligns with the findings of Neijat *et al*. [21], who reported that probiotics derived from other *Bacillus* species had no significant impact on LW and FC in layers. These results suggest that substituting 20% of maize meal with BW is at least as effective as a conventional basal diet in sustaining the health and productivity of laying hens. Similar conclusions were drawn by Fathi *et al*. [22] and Grigorova and Penkov [23], who noted that BW could replace up to 20% of maize meal in laying hen diets without compromising LW or feed efficiency.

Egg production and EM generally increased following dietary supplementation with *B. amyloliquefaciens* CECT 5940 in BW-based diets. Nevertheless, these improvements were not statistically significant compared to the control group. Multiple studies have demonstrated that the efficacy of probiotics can be influenced by several variables, including probiotic strain, dosage, duration of administration, source of isolation, breed of hens, and their physiological stage [21, 22].

Research on the incorporation of *B. amylolique-faciens* CECT 5940 into BW-based diets for laying hens is still scarce. The present investigation contributes to this area by offering empirical evidence supporting the probiotic’s efficacy [24]. Zhou *et al*. [17] and Olafadehan *et al*. [25] found that *Bacillus subtilis* improved egg albumen height and Haugh unit, potentially explaining the observed increase in EM in this study. Consistent with these results, Lei *et al*. [26] reported that the inclusion of *B. amyloliquefaciens* CECT 5940 enhanced both laying performance and egg quality. These authors suggested that *Bacillus* supplementation elevates serum concentrations of follicle-stimulating hormone and estradiol, while reducing levels of stress-related hormones such as adrenocorticotropic hormone and corticosterone, thereby improving both productivity and hen Vb. Furthermore, supplementation with *B. subtilis* C-3102 was found to significantly enhance eggshell strength, thickness, and weight in Boris Brown laying hens [27].

Economic analyses indicated notable reductions in diet costs and overall egg production expenses when *B. amyloliquefaciens* CECT 5940 was added to BW-based diets. Specifically, the cost of feed per kilogram, cost per egg unit, and cost per dozen eggs were significantly reduced. These results support the conclusion that partially replacing maize meal with BW can reduce feed expenditures, thus improving the economic Vb of egg production. Similar cost-saving outcomes were reported by Madiya *et al*. [28], who noted a significant decrease in production costs when bakery waste was used in place of basal diets. As a low-cost by-product, BW offers a financial advantage over maize meal in feed formulations. Epao *et al*. [29] and Osak *et al*. [30] also reported improved profitability in broiler production when bakery waste was incorporated into the diet. Since the choice of feed ingredients substantially influences production decisions [30], it is essential to conduct economic evaluations to determine the impact of alternative feed sources on cost structure and net profitability.

This study presents robust evidence for the economic feasibility of partially substituting maize meal with BW, with or without the inclusion of *B. amyloliquefaciens* CECT 5940, suggesting a viable and cost-effective strategy for poultry producers. Gross revenue per product unit did not differ significantly among the groups, indicating that the inclusion of 20% BW – regardless of probiotic supplementation – does not adversely affect income. Notably, gross value added increased substantially when maize meal was replaced with BW. The addition of *B. amyloliquefaciens* CECT 5940 nearly doubled both the value-added and profitability index relative to the control group (T1). Although the probiotic may incur slightly higher production costs, it is offset by increased revenue from egg sales, resulting in higher profitability. This is further supported by contribution margin analysis, which showed that both T2 and T3 had more favorable margins than T1. In addition, the inclusion of *B. amyloliquefaciens* CECT 5940 significantly improved the break-even point compared to T1, marking a more favorable economic threshold for profitability [28]. These results imply that supplementing BW diets with *B. amyloliquefaciens* CECT 5940 is economically superior to feeding hens a standard basal diet. The improvement is primarily attributed to reduced feed costs and a higher contribution margin, which lowers the number of egg units needed to break even. According to Osak *et al*. [30], a greater contribution margin reduces the break-even point by increasing the income generated per unit sold, whereas a lower margin necessitates higher sales volume to cover fixed costs. Olafadehan *et al*. [25] similarly reported enhanced cost-efficiency in hens-fed bakery waste, underscoring the potential for reducing production costs while simultaneously increasing profitability.

## CONCLUSION

This study demonstrated that the partial replacement of maize meal with 20% BW, with or without supplementation with *B. amyloliquefaciens* CECT 5940, sustained productive performance in laying hens without adverse effects on key zootechnical parameters such as LW, egg-LR, EM, FC/dz, and Vb. While no statistically significant differences were observed in performance metrics across treatments, economic analysis revealed that incorporating BW significantly reduced feed production costs, egg production costs per unit and per dozen, and enhanced gross value added and profitability indices. The inclusion of *B. amyloliquefaciens* CECT 5940 further improved the contribution margin and reduced the break-even point, indicating enhanced economic feasibility.

The strength of this study lies in its comprehensive approach, integrating performance and economic evaluations under practical production conditions, thereby offering a viable, low-cost feeding strategy that leverages agro-industrial waste. In addition, the study provides valuable empirical evidence on the limited but promising role of *B. amyloliquefaciens* CECT 5940 in laying hen nutrition, a relatively underexplored area in poultry science.

However, the study is limited by its relatively short duration (45 days), the use of a single laying hen strain, and the absence of data on egg quality parameters and gut microbiota modulation. Furthermore, the study did not assess the long-term effects of probiotic supplementation or its interaction with other dietary components.

Future research should focus on longitudinal studies that evaluate the impact of BW and *B. amyloliquefaciens* CECT 5940 on egg quality traits, gut health, nutrient digestibility, and immune function across different laying hen breeds and production phases. Investigating the dose-dependent effects of probiotics and their synergy with other functional feed additives would also provide deeper insights into optimizing poultry nutrition while enhancing sustainability and profitability.

## AUTHORS’ CONTRIBUTIONS

AFM, APC, NJM, and CGB: Study conception and design and data acquisition. DHM, LAJ, FDC, and EJC: Literature review and data analysis and interpretation. APC, MG, and CGB: Drafted the manuscript. MG and FDA: Literature review and data analysis and interpretation. FDA, MG, and CGB: Revised the manuscript. All authors have read and approved the final manuscript.
